# T-type channels buddy up

**DOI:** 10.1007/s00424-013-1434-6

**Published:** 2014-01-11

**Authors:** Ray W. Turner, Gerald W. Zamponi

**Affiliations:** 1 Department of Cell Biology and Anatomy, Hotchkiss Brain Institute, University of Calgary, HRIC Bldg, Room 1AA14, 3330 Hospital Dr. N.W., Calgary, T2N 4N1 Alberta Canada; 2 Department of Physiology and Pharmacology, Hotchkiss Brain Institute, University of Calgary, Calgary, T2N 4N1 Alberta Canada

**Keywords:** Cav3, T-type, KCa3.1, KCa1.1, BK, Kv4, A-type

## Abstract

The electrical output of neurons relies critically on voltage- and calcium-gated ion channels. The traditional view of ion channels is that they operate independently of each other in the plasma membrane in a manner that could be predicted according to biophysical characteristics of the isolated current. However, there is increasing evidence that channels interact with each other not just functionally but also physically. This is exemplified in the case of Cav3 T-type calcium channels, where new work indicates the ability to form signaling complexes with different types of calcium-gated and even voltage-gated potassium channels. The formation of a Cav3-K complex provides the calcium source required to activate KCa1.1 or KCa3.1 channels and, furthermore, to bestow a calcium-dependent regulation of Kv4 channels via associated KChIP proteins. Here, we review these interactions and discuss their significance in the context of neuronal firing properties.

## Introduction

Control over the frequency and pattern of neuronal spike output defines neural coding of information in the brain. Central to this process are ion channels that conduct potassium to control excitability by hyperpolarizing the membrane potential. We know of numerous isoforms of voltage-gated potassium channels that contribute to controlling excitability [23, 47]. But few of these have as key a role in regulating the frequency and pattern of spike discharge as calcium-gated potassium channels [135]. A great deal of work has focused on the ability for high-voltage-activated (HVA) calcium channels to activate either small conductance (SK, KCa2.x) [2, 76, 125] or big conductance (BK, KCa1.1) potassium channels to control cell excitability [10–12, 45, 139]. Calcium-dependent control of potassium channels has also been recognized to reflect interactions at the level of either a microdomain or nanodomain, a designation that signifies an interchannel distance of <50 or 50–200 nm, respectively [36]. This is important because it reflects an entirely different degree of control that calcium influx can exert on potassium channel activation that may be necessary to effect different cellular functions. Indeed, interactions at the nanodomain level can allow the voltage dependence of specific HVA calcium channel isoforms to be conferred onto KCa1.1 channels [11], providing greater control over the onset voltage and time of hyperpolarizing currents.

Until recently there were only a few reports of Cav3 (T-type) calcium channels being functionally coupled to activation of either KCa2.x [26, 138] or KCa1.1 [44, 111] channels. This coupling was defined entirely on the basis of physiological interactions with little protein biochemical work to assess the nature of the link or its control of potassium channel function at the level of a microdomain or nanodomain. Recent work reveals that Cav3 calcium channels can form an association at the molecular level with calcium-gated potassium channels and even a voltage-gated potassium channel. Thus, Cav3 channels have been shown through protein biochemical and biophysical analyses to associate closely with KCa1.1 channels [101] as well as intermediate conductance calcium-activated potassium channels (KCa3.1, SK4, KCa3.1) [34]. Moreover, an association at the molecular level was detected between Cav3 channels and the Kv4 family of voltage-gated potassium channels that generate transient A-type currents [4, 5]. In each case linking potassium channel activation to calcium influx through Cav3 channels allows outward current to be triggered from membrane voltages well below resting membrane potential and even over the course of a full-blown spike response. Interestingly, activation of these potassium channels by Cav3 calcium influx relies on three distinct calcium sensing mechanisms. The ability for these complexes to function at either a microdomain or nanodomain level proves to depend on the sensitivity of the calcium sensor in relation to the relatively weak conductance of Cav3 channels compared to HVA calcium channels.

Several recent reviews have been published on the properties of T-type calcium channels on topics that will not be covered here [16, 20, 27, 51, 64, 65, 100, 120, 137]. This review summarizes the current state of knowledge of how Cav3 channel associate with three distinct forms of potassium channel to form ion channel complexes that acquire functional roles that reflect the combination of biophysical properties of each partner in the complex. To fully understand the interplay between calcium and potassium channels, we briefly summarize key features of the responsible subunits and proteins involved.

## Cav3 calcium channels

Voltage-gated calcium channels permit the entry of calcium ions into the cytosol in response to membrane depolarizations. Voltage-gated calcium channels can be divided into two major families: HVA calcium channels that open in response to large membrane depolarizations and low-voltage-activated (LVA) channels that open in response to smaller membrane depolarizations [122]. The HVA channels include L-, P-, Q-, N-, and R-types which can be distinguished based on their biophysical and pharmacological properties. HVA channels share a common multimeric assembly of Cavα1, Cavα2δ, and Cavβ subunits to form a functional complex [17]. Moreover, these channels all interact constitutively with calmodulin [78]. In addition to their more depolarized range of activation, they are distinguished from LVA channels by their larger single channel conductance and open probability, which allow these channels to support large calcium influxes at depolarized potentials. Indeed, it has been estimated that a single HVA calcium channel will increase internal calcium from a resting value of 70–100 nM to greater than 40 μM within a millisecond 15 nm distant from the channel pore [85].

**Fig. 1:**
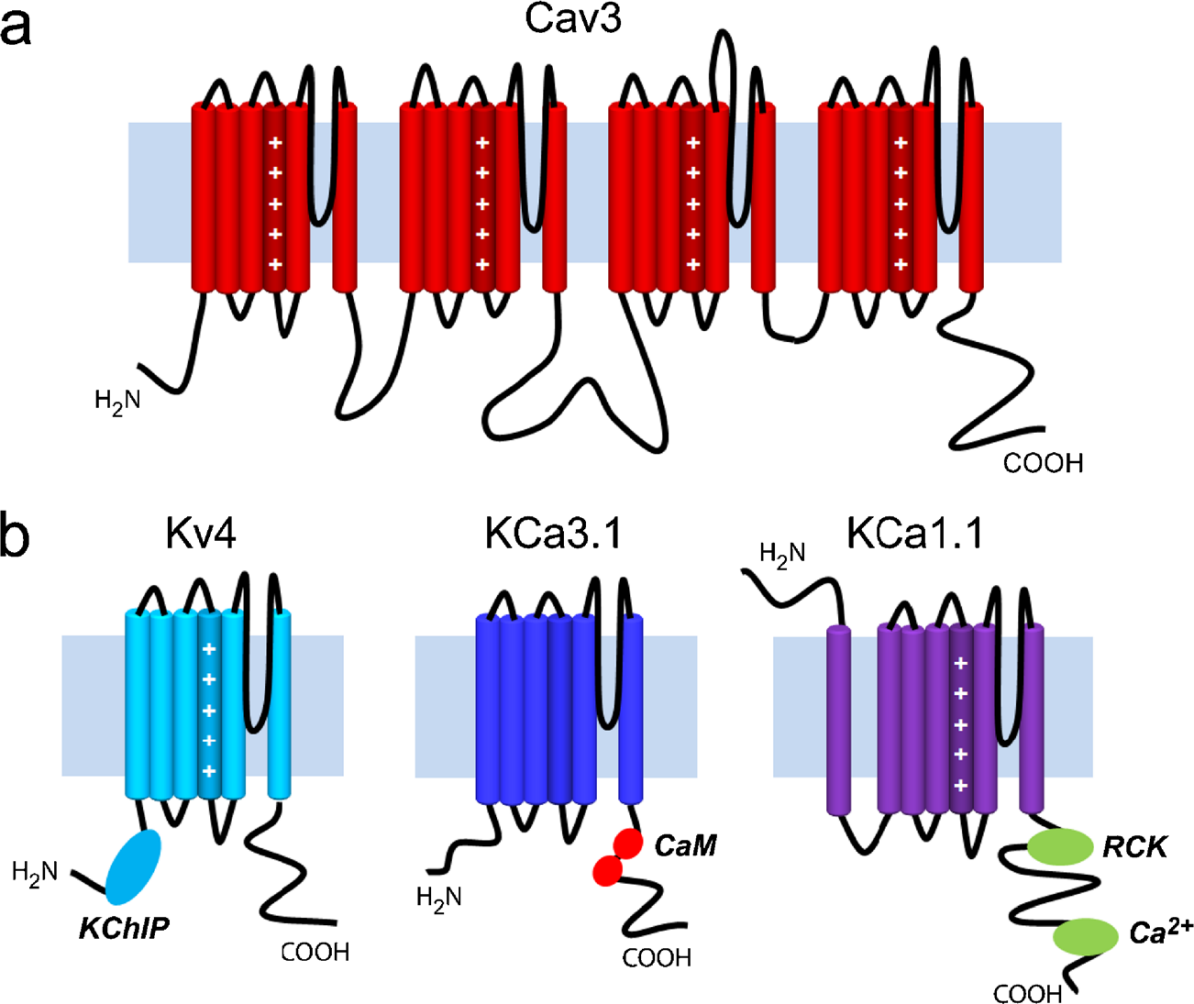
Transmembrane topology of the Cav3 α1 subunit and the three types of potassium channel subunits discussed in this review. **a** Cav3 channels are comprised of four homologous transmembrane domains that are connected via large cytoplasmic linkers. Each of these domains contains six-membrane spanning helices plus a re-entrant pore loop. Voltage sensors are formed by positive charges on the fourth transmembrane helix in each domain. **b** Potassium channel subtypes recognized to form ion signaling complexes with Cav3 calcium channels. Kv4 channels are similar to a single domain of Cav3 channels, and four of these combine to form a functional channel. KChIP molecules attached to the N-terminus region act as calcium sensors. KCa3.1 channels are similar in structure to Kv4 channels but lack voltage sensors. They are purely activated by calcium via interactions with calmodulin (*CaM*) on the channel C-terminus. Finally, KCa1.1 channels show a slightly different membrane topology, with an additional transmembrane helix that places the N-terminus at the extracellular end of the protein. Calcium is sensed by RCK domains and a calcium bowl located on the C-terminus region of the channel

LVA channels encompass the family of T-type calcium channels [97]. These channels are comprised of just a Cavα1 subunit and thus unlike HVA channels do not require assembly with ancillary subunits to replicate native currents. The Cav3 α1 subunit is comprised of four major transmembrane domains that are connected by large cytoplasmic linker regions [97] (Fig. 1a). N- and C-termini are also located on the cytoplasmic side, with sequence variation in the C-terminus or internal linkers being a characteristic delimiter between the Cav3 channel isoforms. All three members of this family (Cav3.1, Cav3.2, and Cav3.3) share a small single channel conductance, rapid activation and inactivation kinetics, and relatively slow deactivation, the latter giving rise to more prolonged calcium influx through tail currents [84]. Furthermore, due to their specific voltage dependencies of activation and inactivation, Cav3 channels give rise to a window current that allows these channels to be tonically active at typical neuronal resting membrane potentials [21, 25, 29, 31, 33, 34, 51]. The voltage range for window current varies between cells but can be anywhere from −90 to +20 mV [31, 33, 34, 118, 121]. The fact that these channels can be tonically active at rest allows them to contribute to functions that range from low threshold exocytosis [136, 137] to spike-activated inward current [118]. Because Cav3 channels are partially inactivated at rest, a membrane hyperpolarization can recover channels from inactivation, giving rise to larger T-type currents during a subsequent depolarization that can directly regulate neuronal firing properties [7, 25, 33, 51, 81, 142]. As we will discuss in this review, Cav3 channels also prove to act as the source of calcium for several different types of potassium channels, thereby supporting an indirect but important means of regulating neuronal firing behavior that was only recently recognized.

## Calcium-gated potassium channels

KCa1.1 and KCa2.x potassium channels have been traditionally recognized as those gated by calcium entry to control membrane excitability in central neurons [1, 12, 129, 135]. The properties of KCa1.1 and KCa2.x channels differ in key respects that support fine-tuned roles in mediating spike repolarization and afterhyperpolarizations (AHPs) over relatively short time frames of activity [1, 66, 67, 85, 116, 123, 133]. All three KCa2.x channel isoforms (KCa2.1–3; SK1–3; KCNN1–3) [1, 12, 135] are purely calcium dependent due to the association of calmodulin with the C-terminal region [1, 57, 58, 63, 141]. While Cav3 calcium channels have been shown to at least functionally couple to KCa2.x channels [26, 138], we will not focus on this given the lack of protein biochemical evidence for a potential ion channel complex at this time.

## KCa1.1

KCa1.1 channels are similar to voltage-gated potassium channels in activating in response to membrane depolarization; however, the voltage dependence of activation is strongly regulated by cytosolic free calcium concentration [28]. KCa1.1 channels are formed by the association of four identical pore forming α-subunits plus ancillary β-subunits which coassemble with the α-subunit in a 1:1 fashion. The α-subunit contains seven membrane spanning helices (S0 to S6), with an extracellular N-terminus and an intracellular C-terminus [28] (Fig. 1b). The C-terminus contains a “regulating conductance of potassium” (RCK) domain and a calcium bowl to confer calcium sensitivity onto KCa1.1 channel activation [32, 43, 46, 82, 102, 111]. The affinity of the calcium binding site on KCa1.1 channels is substantially lower than that of calmodulin associated with KCa2.x and KCa3.1 channels, with reported minimal intracellular calcium concentrations necessary for activation ranging from 1 to >10 μM [12, 85, 123]. As a result KCa1.1 channels are typically activated by relatively large voltage responses such as spike discharge, thereby contributing to spike repolarization and a fast AHP (fAHP) [85, 115, 139]. Previous studies have shown that KCa1.1 channels can form signaling complexes with HVA channels, thereby localizing the channels close to the source of calcium entry [10, 11, 45, 85], but the underlying channel structural determinants are unknown. Indeed, all of Cav1.2 (L), Cav2.1 (P), and Cav2.2 (N) channels can form what were proposed as supercomplexes with KCa1.1 channels, although not with the Cav2.3 calcium channel isoform [10, 12]. The functional importance of this interaction has been repeatedly established, such that blockade of HVA calcium channels (and hence, indirectly, the activity of KCa1.1 channels) in neurons results in drastic alterations of intrinsic neuronal firing properties [109, 112, 115, 117, 139]. New data summarized below now indicates that LVA Cav3 calcium current also has an important role in activating KCa1.1-mediated outward current.

## KCa3.1

KCa3.1 channels are a third class of calcium-gated potassium channel that belong to the same gene family as KCa2.x channels but share only ∼45 % protein sequence homology [52, 57, 72, 135]. KCa3.1 channels are also only calcium dependent through the association with calmodulin [37, 40, 52, 57, 72, 96, 105, 106, 113] but activate and deactivate over a much longer time frame (up to seconds) than KCa1.1 or KCa2.x channels [50, 68, 69, 126]. KCa3.1 channels are derived from a single gene (KCNN4) with virtually all KCa3.1 channels sequenced from various body tissues (i.e., pancreas, placenta, lymphocytes) sharing an equivalent sequence [52, 57, 72]. KCa3.1 channels have a six-transmembrane domain structure and intracellular N- and C-termini (Fig. 1b). The proximal C-terminus contains a constitutive binding site for calmodulin [58] with an IC_50_ for calcium from 95 to 300 nM compared to ∼300–500 nM for KCa2.x channels [1, 57], making KCa3.1 channels potentially more sensitive to changes in internal calcium concentration.

The expression pattern and properties of KCa3.1 channels have been extensively examined outside the CNS, where KCa3.1 channels are expressed in red blood cells [41], endo- and epithelial cells [8, 39, 48, 119, 130, 132], lymphocytes [42, 61], and glia [19, 60, 61, 71]. The function of KCa3.1 channels in controlling neuronal excitability has been best characterized in enteric neurons, where KCa3.1 channels generate a slow AHP (sAHP) of seconds duration [91, 92, 128]. However, in central regions, KCa3.1 channels were not believed to be expressed in neurons [52, 53, 72] but instead were restricted to endothelial cells and activated glia [9, 53, 124, 140]. Nevertheless, reports suggesting the expression of KCa3.1 channels in more central neurons exist, with molecular, immunocytochemical, or electrophysiological data reported in sensory cells of peripheral and autonomic nervous systems [39, 83, 90, 92, 127] as well as motor neurons [13, 83]. Most recently, KCa3.1 channel expression in a central neuron was established for the first time in cerebellar Purkinje cells [34], raising questions as to how widespread KCa3.1 channel expression may be in other central regions. Moreover, the first study examining KCa3.1 channels in Purkinje cells revealed the presence of a Cav3-KCa3.1 complex with important functional roles [34].

## Voltage-gated A-type potassium channels

Among the myriad of voltage-gated potassium channels are a small subset of channels that are activated from a low membrane voltage and in a transient fashion to trigger “A-type” currents. In central regions, A-type potassium currents are often generated by members of the Kv4 channel family (Kv4.1–4.3; KCND1–3) [54, 108]. As found for Cav3 calcium channels, Kv4 channels exhibit a low voltage for activation, fast inactivation, near-complete inactivation at resting potential, and an availability that is governed by preceding membrane hyperpolarizations [54]. Kv4 channels share the common structure of potassium channels in being comprised of four α-subunits containing six transmembrane domains, an S4 voltage-sensing domain, and intracellular C- and N-termini (Fig. 1b). All structural indices then suggest that Kv4 channels belong entirely to the class of voltage-gated channels. However, reports as early as the 1980s began to suggest that A-type potassium channels may also be regulated by calcium influx, although the mechanism was never resolved [14, 18, 74, 107, 143]. Kv4 channels were subsequently found to link to “potassium channel interacting proteins” (KChIP1–4; Fig. 1b), a class of calcium sensor molecules that affect channel translocation and kinetic properties [3, 15, 62, 94, 98, 104, 110, 131]. A second auxillary subunit termed dipeptidyl peptidase-like proteins (DPPs) was identified as membrane spanning proteins that directly interact with the Kv4 α-subunit [30, 38, 54, 56, 59, 87, 88, 99, 103]. While DPPs also exert important effects on baseline properties of Kv4 channels, there is no evidence to date for a role in mediating calcium-dependent effects. The combination of Kv4 α-subunits together with KChIP and DPP proteins together is now recognized as comprising the “Kv4 complex” [22, 24, 55, 59, 75, 86].

The presence of KChIP molecules as part of the Kv4 complex signifies the presence of a very different form of calcium sensor than those inherent to KCa3.1 or KCa1.1 channels. Here, four KChIP molecules bind to the Kv4 α-subunit N-termini to form a “cross-shaped octomer” on the cytoplasmic side to bind calcium [98, 131]. KChIPs1–4 are derived from separate genes and belong to the larger family of calcium sensor proteins that contain four EF-hand domains on the C-terminus [15, 73]. EF-1 does not bind calcium and EF-2 is bound to magnesium, leaving EF-3 and EF-4 as potential calcium binding sites. The dissociation constant for calcium binding to EF-3 and EF-4 is 5 μM [95] and therefore midrange in sensitivity to [Ca]_i_ from that of calmodulin (<300 nM) or KCa1.1 RCK domains (1–10 μM). Although KChIP proteins were identified as a mechanism to provide calcium-dependent regulation of Kv4 current, the effects of calcium influx on KChIPs and Kv4 current were not subsequently reported. The potential source of calcium that might activate KChIPs also remained unknown. As elaborated below, recent work reveals that Cav3 calcium channels selectively associate with the Kv4 complex to modulate A-type current in a KChIP3-dependent manner [4, 5].

## Cav3 interactions with potassium channels

### Cav3.2-KCa3.1 complex

**Fig. 2:**
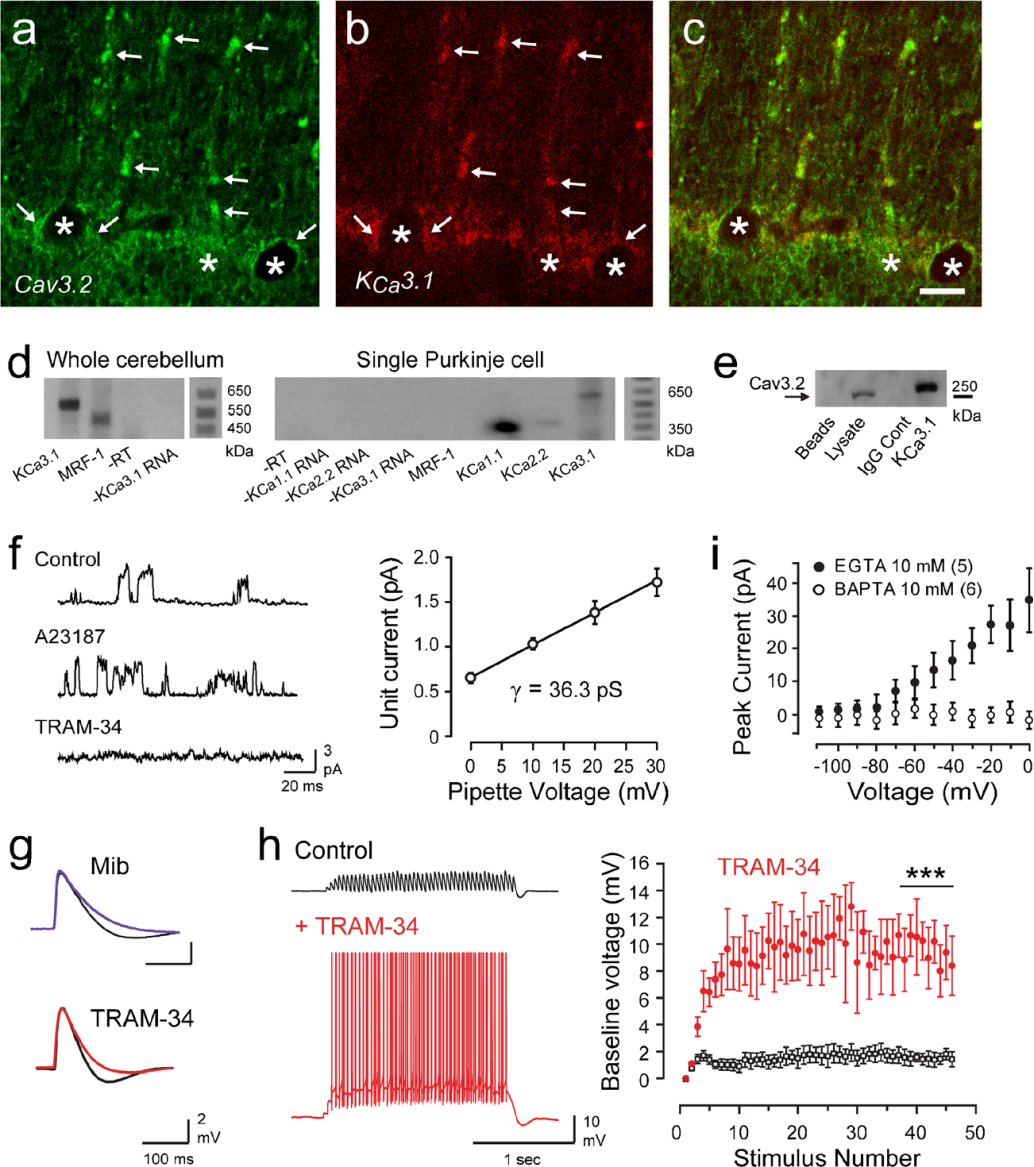
Cav3 calcium channels associate with KCa3.1 channels to regulate temporal summation of EPSPs. **a**–**c** Dual-label immunocytochemistry for Cav3.2 (**a**) and KCa3.1 (**b**) reveals protein colocalized (*arrows*) at the soma (*asterisks*) and restricted segments of dendrites (**c**). **d** RT-PCR reveals KCa3.1 and MRF-1 mRNA in the whole cerebellum (*left*) and KCa1.1, KCa2.2, and KCa3.1 but not MRF-1 in single Purkinje cell cytoplasmic extracts (*right*). **e** KCa3.1 protein coimmunoprecipitates with Cav3.2 from rat cerebellar lysate. **f** On-cell channel recordings (+30-mV pipette potential) before and after perfusing the calcium ionophore A23187 (2 μM) and blocked by TRAM-34 (100 nM). Plot of mean single channel amplitudes at steady-state potentials up to +30 mV reveals a mean conductance of 36.3 pS (*n* = 5). **g** Injection of simEPSCs in Purkinje cells reveals that the simEPSP rate of decay is reduced by mibefradil (1 μM) and TRAM-34 (100 nM). **h** Recordings and plots of the baseline membrane voltage during 25-Hz trains of parallel fiber-evoked EPSPs and TRAM-34 perfusion show that IKCa channels suppress temporal summation of EPSPs and repetitive spike output. **i** Mean values of TRAM-sensitive current in outside-out patches reveal a block of the Cav3-IKCa interaction with internal BAPTA but not EGTA. *Scale bar* in (**a**–**c**) 20 μm. Mean ± SEM; ***p < 0.001. *Abbreviation*: *Mib* mibefradil. Modified from [34]

A recent study on the ionic basis for a sAHP that follows parallel fiber-evoked excitatory postsynaptic potentials (EPSPs) in rat cerebellar Purkinje cells came to the conclusion that KCa3.1 channels were in fact expressed in these cells and activated by forming a complex with Cav3.2 calcium channels [34]. Thus, dual immunocytochemistry revealed KCa3.1 in the somatic region of Purkinje cells and on isolated segments of primary dendrites of these cells (Fig. 2a–c). Moreover, KCa3.1 immunolabel was strongly colocalized with that of Cav3.2 in both somatic and dendritic locations (Fig. 2c). The validity of the immunolabel was supported by RT-PCR in which KCa3.1 signal was detected on Western blots from lysates of whole cerebellum as well as cytoplasmic extracts obtained through whole-cell patch recordings in vitro to conduct single-cell RT-PCR (Fig. 2d). Important controls included the detection of bands for KCa1.1 and KCa 2.2, but not for microglial response factor-1 (MRF-1), a specific marker for microglia, thus confirming that the cytoplasmic sample did not include IKCa3.1 mRNA from nearby microglia. KCa3.1 protein proved to coimmunoprecipitate with Cav3.2 from rat cerebellar lysate, again indicating a close association between these two channels (Fig. 2e). The presence of intermediate conductance potassium channels was confirmed through on-cell single channel recordings from Purkinje cell somata (Fig. 2f). Subsequent addition of the calcium ionophore A23187 greatly increased the open probability of the single channels, which were blocked by perfusion of TRAM-34, a lipophilic compound that enters the cell to block the channel at a site near the inner pore (Fig. 2f). Measuring unit current amplitude over a series of on-cell pipette voltage steps revealed that the single channel conductance was ∼36 pS, a value consistent with that previously reported for KCa3.1 channels (Fig. 2f) [52].

The physiological role for KCa3.1 channels became apparent when simulated EPSCs (simEPSCs) were used to evoke simulated EPSPs (simEPSPs) at the level of Purkinje cell somata to test the role of postsynaptic ion channels in generating a synaptic response [34]. Applying either mibefradil to block Cav3 calcium entry or TRAM-34 to block KCa3.1 channels produced an equivalent block of the decay phase of the simEPSP in Purkinje cells (Fig. 2g). By comparison, none of the established blockers for HVA calcium channels, KCa2.x channels, or KCa1.1 channels could reproduce these effects. When a train of subthreshold simEPSPs was applied at 25 Hz, it was found that temporal summation of EPSPs was strongly suppressed beyond the first four to five stimuli but that consistent and continual spike discharge was evoked when TRAM-34 was applied to block KCa3.1 channels (Fig. 2h). Importantly, TRAM-34 was also effective in the presence of picrotoxin used to block feedforward inhibitory projections activated by parallel fibers [79], indicating that both systems serve to regulate EPSP summation. KCa3.1 channel activation by Cav3 calcium influx could thus be traced to suppressing the baseline EPSP-membrane voltage response during repetitive synaptic input, acting as a high-pass filter to suppress background parallel fiber synaptic activity under normal conditions.

The link between Cav3.2 calcium influx and KCa3.1 activation was all supported by the close association indicated through coimmunoprecipitation and immunocytochemistry. To further assess the distance between the two channels in the complex, we employed the use of the two calcium chelator EGTA and BAPTA. Here, it was shown in outside-out patch recordings that TRAM-34-sensitive outward current was present when 10 mM EGTA was included in the electrode but not in the presence of 10 mM BAPTA (Fig. 2i). This was important in establishing that the Cav3-KCa 3.1 complex functions at the level of a calcium nanodomain or <50-nm distance [36]. The exact sites that form a link between Cav3.2 and KCa3.1 have not yet been identified nor has the degree to which these findings extend to Cav3.1 or Cav3.3. Yet taken together, these data argue strongly for the first nanodomain interaction between Cav3 calcium channels and a calcium-activated potassium channel.

### Cav3.2-KCa1.1 complex

**Fig. 3:**
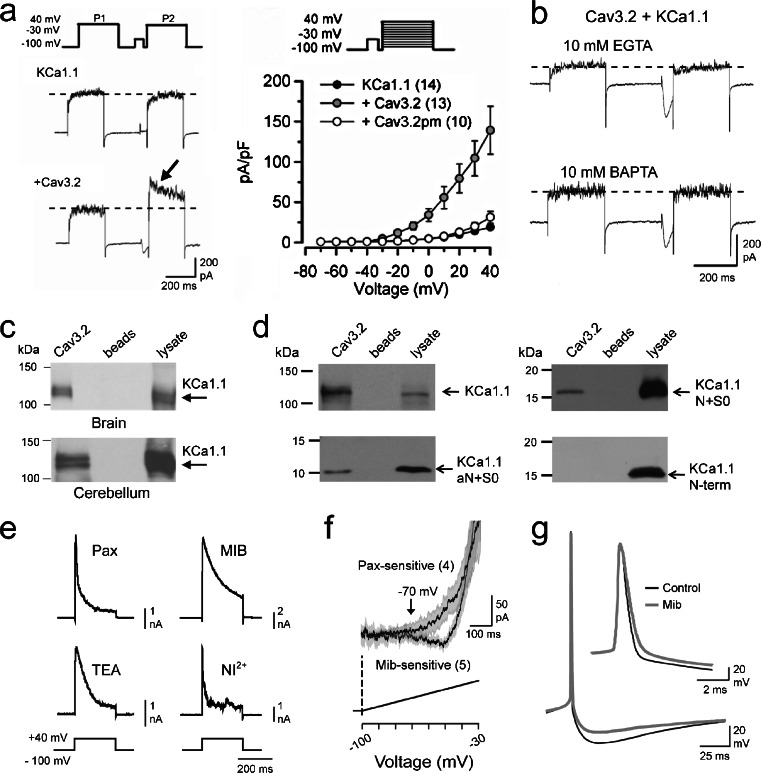
A Cav3.2-KCa1.1 channel complex generates a LVA KCa1.1 current involved in spike repolarization and a fAHP. **a** Whole-cell patch recordings from tsA-201 cells expressing KCa1.1 and/or Cav3.2 cDNA reveal that Cav3.2 calcium influx triggered by a prepulse command augments KCa1.1 current (*arrow*). Steps: 250 ms, +40 mV; prepulse 50 ms, −30 mV, return 2 ms. Plots of mean current for KCa1.1 expressed alone, with Cav3.2, or a noncalcium-conducting Cav3.2 pore mutant (Cav3.2pm). **b** KCa1.1 current augmentation by Cav3 calcium influx during a step prepulse in tsA-201 cells is blocked by internal EGTA or BAPTA. **c** Cav3.2 and KCa1.1 proteins coimmunoprecipitate from lysates of the rat brain or cerebellum. **d** Lysates from tsA-201 cells reveal that Cav3.2 coimmunoprecipitates with full-length KCa1.1, N + S0, and aN + S0, but not the KCa1.1 N-terminus expressed in isolation. Western blots reflect myc-tagged KCa1.1 channels or their fragments. **e** Whole-cell currents in MVN cells isolated as paxilline (1 μM)- or TEA (1 mM)-sensitive KCa1.1 current compared to currents isolated as mibefradil (1 μM)- or Ni^2+^ (300 μM)-sensitive (Cav3-activated) currents. **f** Average plots of currents in MVN cells isolated by either paxilline (1 μM) or mibefradil (1 μM) during ramp commands (SEM values *shaded*). **g** Mibefradil (1 μM) slows spike repolarization and reduces the fAHP in a MVN cell. Mean ± SEM. *Abbreviations*: *Mib* mibefradil, *pax* paxilline. Modifed from [101]

A second interaction between Cav3 channels and a calcium-activated potassium channel was recently reported for KCa1.1 channels [101]. Here, the relationship between Cav3 calcium influx was examined through expression of cDNAs in tsA-201 cells and in cells of the rat medial vestibular nucleus (MVN) maintained as an in vitro slice preparation. Coexpressing cDNAs for human Cav3.2 and KCa1.1 in tsA-201 cells revealed that while a voltage command pulse delivered from a value of −100 to +40 mV did not reliably activate KCa1.1 current, preceding this pulse with a step to −30 mV to maximally activate Cav3 current augmented KCa1.1 current (Fig. 3a). The mechanism for KCa1.1 activation was apparent in *I*-*V* plots where a Cav3 calcium prepulse left-shifted the voltage dependence for KCa1.1 activation, a result expected due to the interplay between calcium- and voltage-gated activation of KCa1.1 channels. Corroboration of the role for Cav3-mediated calcium influx was obtained when expressing KCa1.1 cDNA with a Cav3.2 pore mutant that does not conduct calcium (Cav3.2pm) prevented the leftward shift in KCa1.1 voltage for activation (Fig. 3a). Notably, the Cav3-mediated effect proved to be sensitive to block not only by internal perfusion of BAPTA but also by as little as 5 mM EGTA (Fig. 3b), the implications of which are discussed further below.

Protein biochemical tests revealed that Cav3.2 channels coimmunoprecipitate with KCa1.1 from lysates of either the brain or cerebellum (Fig. 3c) and from lysates of tsA-201 cells expressing only the channel α-subunits (Fig. 3d). Further tests for coimmunoprecipitation between Cav3.2 and different KCa1.1 mutant channels further narrowed down the site for interaction. Thus, Cav3.2 channels coimmunoprecipitated with a mutant construct comprised of only the KCa1.1 N-terminus and S0 transmembrane segment (N + S0), with a naturally occurring truncated N-terminal variant with SO (aN + S0), but not with a construct comprised of only the longer N-terminus (Fig. 3d). These data strongly implicate a site of interaction between Cav3.2 and the transmembrane S0 segment.

Previous work had reported at least a functional coupling between Ni^2+^-sensitive calcium influx and KCa1.1 in MVN cells [111]. However, direct biophysical tests of the nature of this activation had not been reported. We used whole-cell voltage clamp of MVN cells in vitro to examine KCa1.1 current isolated by either paxilline (1 μM) or TEA (1 mM) application and compared these to outward currents isolated by perfusing the Cav3 blockers mibefradil (1 μM) or Ni^2+^ (300 μM) (Fig. 3e) [101]. In each case the isolated current was fast activating and slowly inactivating over the time of a 200-ms step command to +40 mV (Fig. 3e). Using a ramp command over a −100 to −30 mV range with paxilline or mibefradil treatment further revealed LVA outward current that activated just subsequent to LVA inward calcium current, indicating that Cav3 calcium influx can activate KCa1.1 above ∼−70 mV (Fig. 3f). Finally, applying mibefradil to MVN cells under current clamp slowed spike repolarization, reduced a subsequent fAHP (Fig. 3g), and increased the gain of firing, revealing that the Cav3-KCa1.1 interaction normally reduces spike output in MVN cells. These data were entirely novel in identifying a new association between Cav3 and KCa1.1 channels in an ion channel complex, helping account for earlier reports of the role for KCa1.1 in controlling gain in MVN cells [89, 111].

### Cav3-Kv4 complex

A series of recent studies on stellate cell interneurons in the cerebellum led to a new understanding of how Cav3 channels can interact with even voltage-gated potassium channels. Earlier work on the expression pattern of Cav3 channel isoforms in these cells [77] led to the finding that membrane hyperpolarizations expected to promote a rebound increase in firing frequency instead promoted a unique voltage-dependent shift in first spike latency [80]. As ionic control of first spike latency has traditionally been assigned to A-type potassium channels, it prompted further investigation into how Cav3 channels might influence this important aspect of spike firing dynamics. It was finally concluded that the coexpression of Cav3 and Kv4 channels led to a non-monotonic profile in the voltage-first spike latency profile, with Cav3 calcium influx reducing latency from more hyperpolarized potentials, and Kv4 current increasing spike latency from membrane potentials near rest [80]. However, there was a curious peak in the voltage-latency profile near resting membrane potential that proved to be sensitive to Cav3 channel blockers, implying that Cav3 and Kv4 channels may establish first spike latency characteristics by interacting at a level beyond simple coexpression in the membrane.

**Fig. 4:**
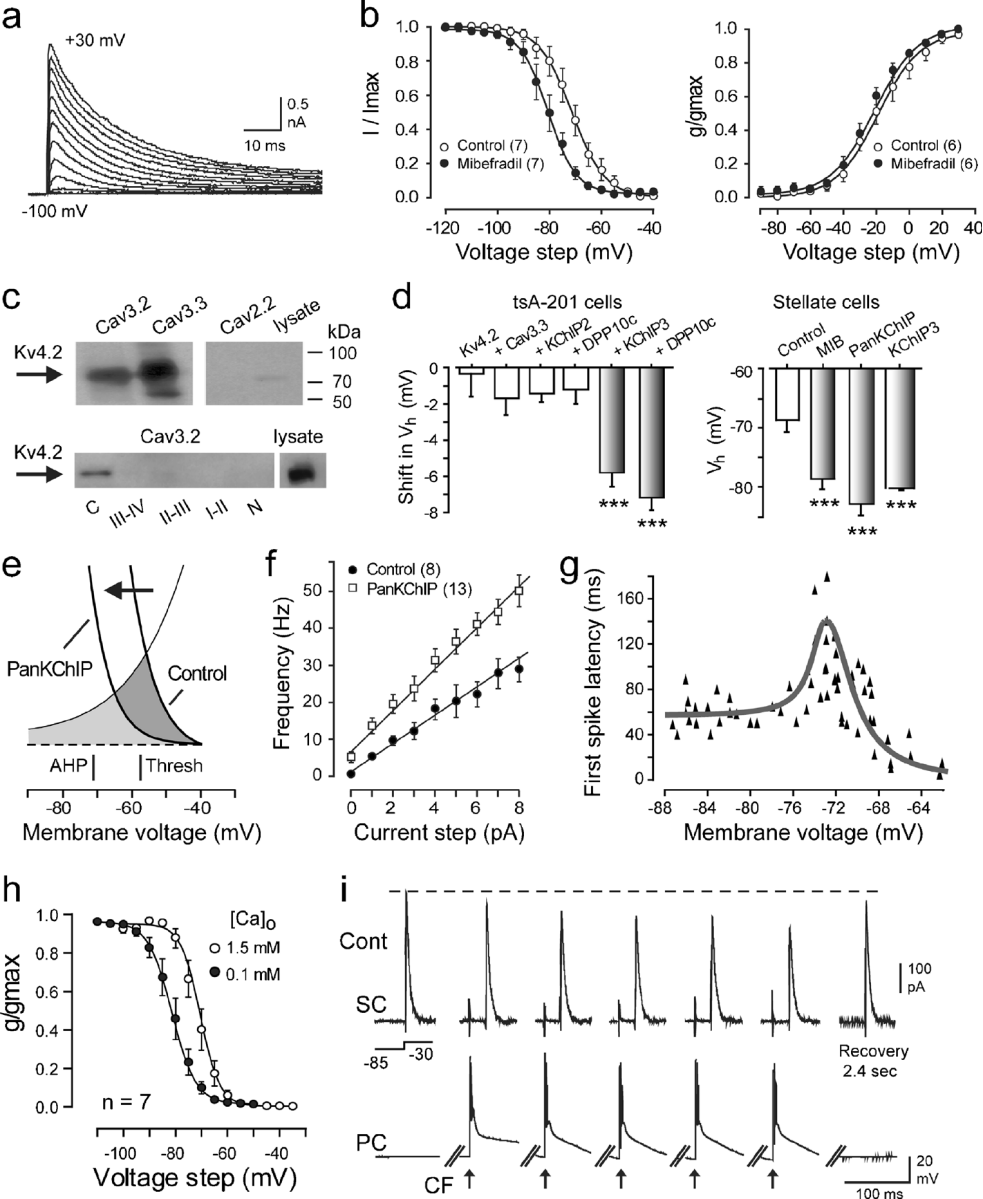
A Cav3-Kv4 complex in stellate cells regulates *I*_A_ availability near rest and acts as an extracellular calcium sensor. **a** Whole-cell voltage clamp of *I*_A_ in cerebellar stellate cells in the absence of calcium channel blockers. **b** Blocking Cav3 calcium influx with mibefradil (0.5 μM) selectively left-shifts the voltage-inactivation plot (*V*_h_) ∼−10 mV without affecting voltage for activation. **c** Kv4.2 channel coimmunoprecipitates with Cav3.2 or Cav3.3 protein but not Cav2.2 from rat brain lysate (*top row*) and pulls down with the C-terminus of Cav3 GST fusion proteins (*bottom row*). **d** Coexpressing different Kv4 complex members in tsA-201 cells reveals a critical role for KChIP3 in mediating a mibefradil-induced shift in Kv4 *V*_h_ (*left*). Internal perfusion of PanKChIP or KChIP3 antibodies into stellate cells selectively disrupts the Cav3-Kv4 complex function to induce the same shift in *V*_h_ as mibefradil (*right*). **e** Magnified view of the foot of Kv4 activation and inactivation plots indicating that a leftward shift in *V*_h_ (*arrow*) by dialyzing a PanKChIP antibody reduces window current in the region of spike threshold. **f**, **g** Plots showing the role of the Cav3-Kv4 complex in normally reducing firing rate gain (**f**) and increasing first spike latency near resting potential (**g**) in stellate cells in response to current pulse injections. **h** Dependence of the Cav3-Kv4 complex function on calcium influx enables sensitivity to decreases in [Ca]_o_. Test pulse −30 mV. **i** Dual recordings of *I*_A_ in a cerebellar stellate cell and complex spike discharge in a Purkinje cell directly below the stellate cell recording. Repetitive 10-Hz CF stimulation and complex spike discharge is associated with a decrease in *I*_A_ (*dashed line*). Mean ± SEM; *Abbreviations*: *Thresh* threshold, *SC* stellate cell, *PC* Purkinje cell, *CF* climbing fiber. ***p < 0.001. Modified from [4, 6, 80]

Further studies on this potential interaction departed from the standard procedure of blocking all calcium influx when isolating potassium channels and found that under conditions of blocking sodium and HCN channels, the prominent current recorded was a transient Kv4 A-type response (Fig. 4a) [4]. However, blocking Cav3 calcium influx with mibefradil (0.5 μM) revealed a select leftward shift in the voltage-inactivation profile for Kv4 current (*V*_h_) without any effect on the voltage for activation (Fig. 4b). Importantly, these effects were not due to direct effects on the channel, as no such influence was detected on Kv4 channels expressed in isolation in tsA-201 cells. Protein biochemical studies on Cav3.2 and Cav3.3 channel isoforms (those inherently expressed in stellate cells) further established that Cav3 (but not Cav2.2) proteins coimmunoprecipitated with Kv4.2 from brain lysates (Fig. 4c). Moreover, Kv4.2 channels selectively pulled down with the C-terminal region of Cav3.2 or Cav3.3 channels among a set of glutathione *S*-transferase (GST) fusion proteins of each of the internal Cav3 channel linkers and C- and N-termini expressed in tsA-201 cells (Fig. 4c). These results are important in establishing that Kv4.2 channels form a direct molecular association with the C-terminal region of Cav3 channels. In fact, subsequent work established that this link can be detected between all Kv4 isoforms and all Cav3 channel isoforms [5]. In further characterizing the basis for Cav3-Kv4 function, different combinations of Kv4 complex subunits were expressed in tsA-201 cells to identify the minimal complement necessary to reproduce a mibefradil-induced shift in Kv4 *V*_h_. This work showed that expressing a Cav3 and Kv4 channel isoform is necessary but that the KChIP3 isoform is necessary and sufficient as an accessory protein to account for calcium-dependent regulation of Kv4 *V*_h_ (Fig. 4d) [4]. By comparison, none of the HVA calcium channels (P/Q-, N-, L-, or R-type) or other KChIP isoforms were able to modulate Kv4 current when tested in tsA-201 cells. These data are important in showing that only the Cav3 calcium channel can associate with the Kv4 channel complex to confer calcium-dependent control through the specific calcium sensing action of KChIP3.

A series of studies established that infusing either a KChIP3 or PanKChIP antibody through the electrode (1:100 dilution) during stellate cell recordings mimicked the effects of applying mibefradil in left-shifting Kv4 *V*_h_ (Fig. 4d), providing the first means of interfering with Cav3-Kv4 complex function. With this tool in hand, one could test the functional role of a Cav3-Kv4 complex, with antibody infusion revealing that a Cav3 calcium-mediated rightward shift in the voltage-inactivation profile ordinarily acts to increase Kv4 window current in the region of spike threshold (Fig. 4e). As a result, the Cav3-Kv4 complex reduces cell excitability, as measured by a significant drop in firing rate gain (Fig. 4f) [4]. A Cav3-mediated increase in Kv4 window current near rest also accounted for the increase in the voltage-first spike latency relationship that was identified in stellate cells near resting potentials (Fig. 4g) [80]. While repetitive stimulation to increase internal calcium concentration is without apparent effect on Kv4 availability, the Cav3-Kv4 complex proves to be highly sensitive to decreases in extracellular calcium levels (Fig. 4h) [6]. This is important because it has been established that physiological rates of excitatory afferent input to the cerebellum rapidly decrease extracellular calcium concentration in the order of 30 % from resting values [49, 93, 114]. We thus used dual recordings between a Purkinje cell and a stellate cell directly above in the molecular layer to assess the effects of climbing fiber stimulation on *I*_A_ and *I*_T_ [6]. As shown in Fig. 4i, *I*_A_ was isolated pharmacologically while recording from a Purkinje cell under current clamp to monitor complex spike discharge. Repetitive complex spike discharge at 10 Hz rapidly decreased *I*_A_ amplitude, with full recovery within 2–3 s after the end of a stimulus train. Similarly, climbing fiber stimulation decreased *I*_T_ in stellate cells during a stimulus train, with even faster kinetics of recovery (data not shown). Since the decrease in *I*_A_ availability increased the firing rate gain of stellate cells, it was found that the Cav3-Kv4 complex functions to provide adaptive inhibitory control over Purkinje cells in the face of excitatory afferent-induced reductions in extracellular calcium [6]. These findings were key in establishing that expression of the Cav3-Kv4 complex in stellate cells ultimately underlies a new form of homeostatic control over circuit function [6].

## Microdomain versus nanodomain interactions

Given widely different calcium sensors and potassium channels involved in these ion channel complexes, it is interesting to compare the relative efficacy of Cav3 calcium channels to effect potassium channel activation. Previous work has shown that calcium influx gives rise to a domain of decreasing internal calcium surrounding the internal pore of the channel. The distance over which calcium influx can trigger a calcium-dependent event is distinguished by those functioning at the level of a calcium microdomain (50–200-nm distance) or a nanodomain (<50 nm) [36]. It is known that interactions between the HVA calcium channels Cav2.1 or Cav2.2 and KCa1.1 in an expression system occur at the level of a nanodomain [11]. The ability for HVA calcium influx to activate KCa1.1 is such that even the voltage dependence and kinetics of either calcium channel subtype is imparted upon KCa1.1 activation. Given that all of the complexes reviewed here share Cav3 channels as a common partner, key factors that could be predicted to control the degree of potassium channel activation would be (1) the relative proximity of calcium and potassium channels, (2) the sensitivity of the calcium sensors to changes in internal calcium, and (3) the relative conductance of Cav3 calcium channels. The latter is of particular interest given that Cav3 channels have a much lower conductance than HVA calcium channels and exhibit rapid inactivation that will substantially reduce the time for internal calcium accumulation.

**Fig. 5:**
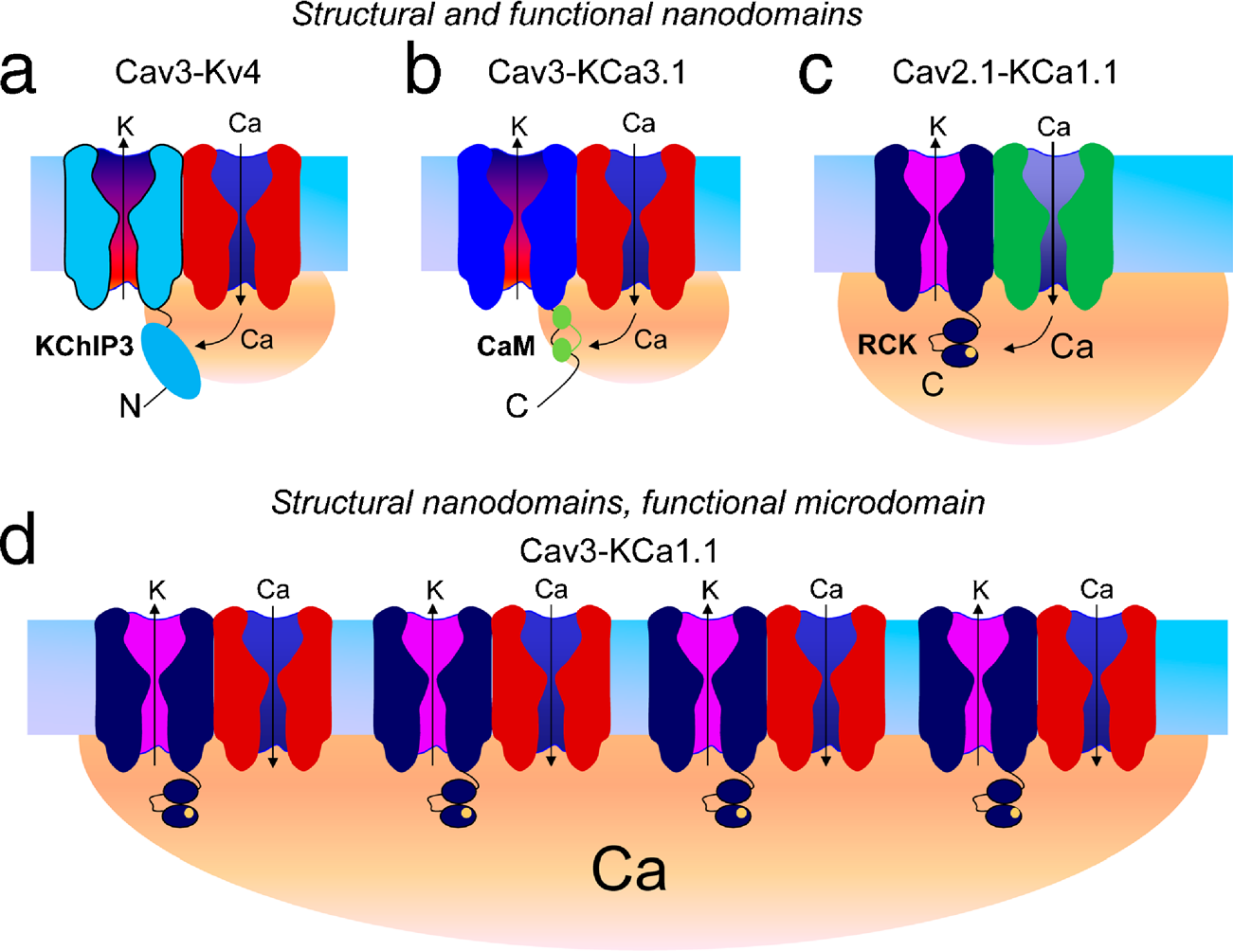
Interactions between calcium channels and potassium channels. Shaded regions (*orange*) represent the size of calcium domains. **a** Cav3 channels physically associate with Kv4 channels, placing the Kv4 channel and its associated KChiIP3 accessory protein within a calcium nanodomain formed by calcium entering through the pore of an individual Cav3 channel. **b** Similarly, KCa3.1 channels associated with a Cav3 channel are placed within this nanodomain, and the high calcium affinity of calmodulin ensures that KCa3.1 channels can be gated by an individual Cav3 channel. **c** KCa1.1 channels require a higher calcium concentration for activation. In the case of N-type channels, the higher open probability and greater single channel conductance than Cav3 channels is sufficient to activate an associated KCa1.1 channel. **d** In contrast, individual Cav3 channels do not provide sufficient calcium to activate KCa1.1 channels, instead requiring the concerted action of multiple Cav3-KCa1.1 complexes to raise calcium to sufficiently high levels to permit KCa1.1 activation, accounting for the EGTA sensitivity of this process despite the physical association of the channels

The ability to coimmunoprecipitate Cav3 channels and either KCa3.1 or Kv4 channels is consistent with the physical requirements expected for a nanodomain interaction. In both cases the calcium-induced change in potassium channel function is prevented by internal perfusion of BAPTA but not EGTA. Moreover, the voltage-dependent and kinetic properties of Cav3-mediated calcium influx are transferred to KCa3.1 channels to produce a LVA transient outward current [34]. The potential conveyance of Cav3 voltage-dependent properties on Kv4 channels is less readily discerned given the similarities between these two channels in this parameter. However, the ability for calcium to modify the voltage-inactivation profile of Kv4 channels signifies the ability for the complex to convert a voltage-gated Kv4 channel to one that is dependent on calcium influx. In fact, without Cav3 calcium influx, the leftward shift in Kv4 *V*_h_ would substantially increase Kv4 inactivation at resting membrane potentials. Together, these data support the conclusion that Cav3 channels are positioned sufficiently close to KCa3.1 or Kv4 channels, and the sensitivity of calmodulin and KChIP3 as calcium sensors is sufficiently high to support a nanodomain interaction even with the relatively low conductance of Cav3 channels (Fig. 5a, b). Interestingly, the Cav3-Kv4 complex proves to be so highly sensitive to Cav3 calcium conductance that even subtle reductions in extracellular calcium during physiologically relevant input signals reduce *I*_A_ availability [6]. However, this sensitivity is valuable in allowing the complex to act as a novel sensor for extracellular calcium to modify stellate cell output in an adaptive manner.

In the case of KCa1.1 channels, the situation is less clear. On the one hand, coimmunoprecipitation data indicate that Cav3 and KCa1.1 channels are part of a macromolecular signaling complex that places KCa1.1 channels close to the source of Cav3 calcium entry. But Cav3-mediated activation of KCa1.1 is less reliable in being blocked by as little as 5 mM internal EGTA in both tsA-201 and MVN cells. Activating KCa1.1 in tsA-201 cells further required an initial prepulse command to −30 mV to maximally activate Cav3 current. Once sufficient activation of KCa1.1 was achieved, Cav3 calcium influx left-shifted the voltage dependence of KCa1.1 activation, with additional evidence for voltage-dependent inactivation of KCa1.1, as expected for steady-state inactivation properties of Cav3 channels. These data are perplexing in presenting a calcium-dependent activation process more reminiscent of a microdomain interaction. However, this implies a distance between the channels of 50–200 nm, which is greater than what one expects from a direct molecular interaction. In contrast, it has been shown that physical signaling complexes between N-type calcium channels and KCa1.1 channels allow a functional interaction at the level of a nanodomain (Fig. 5c). The key difference from Cav3 channels is a much larger open probability and single channel conductance of N-type channels, such that each channel is sufficient to provide the calcium levels needed for KCa1.1 activation [134].

Lessons learned from studying calmodulin regulation of calcium-dependent inactivation (CDI) of HVA channels can be used to explain this apparent discrepancy. For both L-type and P/Q-type channels, calmodulin is preassociated with the calcium channel. A rise in intracellular calcium results in calcium binding to calmodulin, thereby triggering a rearrangement of the channel-calmodulin complex to cause CDI [35]. Calmodulin has two high-affinity and two low-affinity binding sites for calcium. CDI of L-type channels is dependent on the high-affinity calcium binding lobes, whereas that of P/Q-type channels relies on the low-affinity binding sites. Therefore, calcium entry via an individual L-type channel is sufficient to raise calcium concentrations near the high-affinity sites on calmodulin to trigger CDI, making this resistant to internal EGTA [70]. The situation is different with P/Q-type channels, where the lower affinity of calmodulin for calcium requires a much larger rise in intracellular calcium for CDI to occur. Importantly, this rise in calcium cannot be supported by an individual channel but rather requires the concerted action of many channels [70]. Thus, despite the fact that each channel is already associated with a calmodulin molecule, CDI of P/Q-type channels is EGTA sensitive.

We hypothesize that apparent incongruencies in data showing a Cav3-KCa1.1 macromolecular complex that functions at the level of a microdomain parallel what is observed with CDI of P/Q-type channels. Much like the low-affinity calcium binding site on calmodulin, the calcium binding site on KCa1.1 channels has a low affinity for calcium. Therefore, calcium entry through a single Cav3 calcium channel is insufficient to activate KCa1.1 even though the two proteins are in close proximity. However, a concerted entry of calcium through multiple Cav3-KCa1.1 complexes is proposed to support a global rise in calcium that is sufficiently high to activate KCa1.1 and, thus like CDI of P/Q-type channels, is sensitive to internal EGTA (Fig. 5d). An additional consideration concerns the stoichiometry between Cav3 calcium and an associated potassium channel. Potassium channels are tetrameric assemblies, and thus, in principle, each potassium channel could interact with four Cav3 channels, although experimental evidence for a 4:1 stoichiometry is lacking. Along these lines, each potassium channel may have four different calcium sensors (i.e., four KChiP proteins, four calmodulin molecules, or four RCK domains/calcium bowls). Hence, the calcium dynamics of potassium channel activation/modulation may be immensely complex, giving rise to unexpected EGTA sensitivities that would complicate distance estimates based on the use of intracellular calcium buffers alone.

## Concluding remarks

As we have discussed here, Cav3 channels can form molecular associations with three different potassium channel classes that rely on very different mechanisms for calcium sensing. The primary determinants for functional coupling at either a microdomain or nanodomain level appear to be defined by the relative conductance of the calcium channel and sensitivity of the calcium sensor to changes in intracellular calcium concentration. As result, initial work on the Cav3-KCa1.1 complex revealed a new form of calcium-dependent activation of potassium channels not originally expected according to the conventional means of defining interactions at the micro- and nanodomain levels (i.e., the use of buffer sensitivity alone is insufficient to paint a complete picture). Another novel aspect is the observation that KCa3.1 channels are in fact expressed in CNS neurons where they regulate neuronal output, in part by forming a Cav3-KCa3.1 channel complex that can activate in the subthreshold region. The ability for Cav3 calcium current to activate KCa3.1 channels further confers voltage-dependent gating properties from Cav3 channels onto a normally voltage-insensitive KCa3.1 channel. Conversely, Cav3 channels confer calcium sensitivity onto the normally purely voltage-sensitive Kv4 channels via KChIP3 proteins. This altogether widens the traditional view of different ion channels acting independently—instead, there is intricate interplay between different ionic channels through formation of macromolecular signaling complexes. What remains to be examined is whether there is feedback regulation that allows the associated potassium channels to regulate Cav3 channels. Furthermore, it remains to be determined if the formation of signaling complexes can affect other channel features, such as trafficking to the plasma membrane and specific localization within neurons. Our observations that Cav3-K channel complexes can profoundly impact neuronal output may thus be only one of many important aspects of these molecular associations.
